# HMGB1 neuroimmune signaling and REST-G9a gene repression contribute to ethanol-induced reversible suppression of the cholinergic neuron phenotype

**DOI:** 10.1038/s41380-023-02160-6

**Published:** 2023-07-04

**Authors:** Fulton T. Crews, Rachael P. Fisher, Liya Qin, Ryan P. Vetreno

**Affiliations:** 1 Bowles Center for Alcohol Studies, School of Medicine, University of North Carolina at Chapel Hill, Chapel Hill, NC 27599 USA; 2 Department of Psychiatry, School of Medicine, University of North Carolina at Chapel Hill, Chapel Hill, NC 27599 USA

**Keywords:** Neuroscience, Molecular biology, Cell biology

## Abstract

Adolescent binge drinking increases Toll-like receptor 4 (TLR4), receptor for advanced glycation end products (RAGE), the endogenous TLR4/RAGE agonist high-mobility group box 1 (HMGB1), and proinflammatory neuroimmune signaling in the adult basal forebrain in association with persistent reductions of basal forebrain cholinergic neurons (BFCNs). In vivo preclinical adolescent intermittent ethanol (AIE) studies find anti-inflammatory interventions post-AIE reverse HMGB1-TLR4/RAGE neuroimmune signaling and loss of BFCNs in adulthood, suggesting proinflammatory signaling causes epigenetic repression of the cholinergic neuron phenotype. Reversible loss of BFCN phenotype in vivo is linked to increased repressive histone 3 lysine 9 dimethylation (H3K9me2) occupancy at cholinergic gene promoters, and HMGB1-TLR4/RAGE proinflammatory signaling is linked to epigenetic repression of the cholinergic phenotype. Using an ex vivo basal forebrain slice culture (FSC) model, we report EtOH recapitulates the in vivo AIE-induced loss of ChAT+IR BFCNs, somal shrinkage of the remaining ChAT+ neurons, and reduction of BFCN phenotype genes. Targeted inhibition of EtOH-induced proinflammatory HMGB1 blocked ChAT+IR loss while disulfide HMBG1-TLR4 and fully reduced HMGB1-RAGE signaling decreased ChAT+IR BFCNs. EtOH increased expression of the transcriptional repressor RE1-silencing transcription factor (REST) and the H3K9 methyltransferase G9a that was accompanied by increased repressive H3K9me2 and REST occupancy at promoter regions of the BFCN phenotype genes *Chat* and *Trka* as well as the lineage transcription factor *Lhx8*. REST expression was similarly increased in the post-mortem human basal forebrain of individuals with alcohol use disorder, which is negatively correlated with ChAT expression. Administration of REST siRNA and the G9a inhibitor UNC0642 blocked and reversed the EtOH-induced loss of ChAT+IR BFCNs, directly linking REST-G9a transcriptional repression to suppression of the cholinergic neuron phenotype. These data suggest that EtOH induces a novel neuroplastic process involving neuroimmune signaling and transcriptional epigenetic gene repression resulting in the reversible suppression of the cholinergic neuron phenotype.

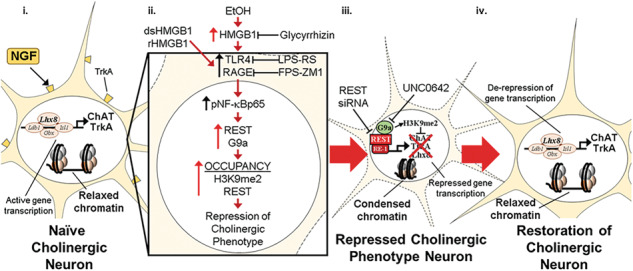

## Introduction

Basal forebrain cholinergic neurons (BFCNs) modulate attention and cognition, and integrate neural networks [1–3] through vast projections to key brain regions, including the cortex and hippocampus [4]. Adolescent binge drinking is associated with increased risk for development of alcohol use disorder (AUD) [5] and neurodegenerative diseases such as Alzheimer’s disease (AD) [6, 7]. Loss of BFCNs occurs in both AUD and AD [8, 9], and is accompanied by induction of proinflammatory neuroimmune signaling molecules, including Toll-like receptor 4 (TLR4), receptor for advanced glycation end-products (RAGE), and the endogenous TLR4/RAGE ligand high-mobility group box 1 (HMGB1) [10–12]. Adolescent intermittent ethanol (AIE) exposure, which models human adolescent binge drinking, causes persistent loss of choline acetyltransferase immunoreactive (ChAT+IR) BFCNs as well as forebrain induction of HMGB1-TLR4/RAGE neuroimmune signaling that persists into adulthood despite cessation of continued ethanol exposure [13–16]. As HMGB1 binds to and activates TLR4, RAGE, and other receptors culminating in nuclear translocation of NF-κBp65 contributing to complex proinflammatory signaling [17], HMGB1 is poised to play a critical role as an immunoregulator underlying neuroimmune signaling and loss of BFCNs. However, alcohol exposure also induces peripheral proinflammatory signaling molecules (e.g., HMGB1, TNFα) that can drive neuroinflammation [18, 19], confounding interpretation of direct HMGB1 neuroimmune involvement in the loss of BFCNs.

Emerging studies link the loss of ChAT+ BFCNs to proinflammatory neuroimmune signaling-induced shifts in chromatin remodeling and gene transcriptional regulation through epigenetic mechanisms that can repress gene transcription [13, 20–23]. Loss of ChAT+IR and other cholinergic neuron markers is generally considered analogous to cell death; however, studies of AIE- and fimbria-fornix lesion-induced loss of ChAT+IR BFCNs find restorative treatments can recover BFCN populations [14, 15, 24], suggesting loss of the differentiated cholinergic neuron phenotype is reversible. Indeed, the in vivo AIE-induced loss of ChAT+IR BFCNs is accompanied by increased occupancy of the transcriptional repressive marker histone 3 lysine 9 dimethylation (H3K9me2) at *Chat* and *Trka* gene promoters that is reversed when ChAT+ BFCNs are restored [14, 15]. In the current study, we used an ex vivo basal forebrain slice culture (FSC) model to test the hypothesis that EtOH-induced HMGB1-mediated neuroimmune signaling causes epigenetic repression of the BFCN phenotype. We report that EtOH exposure induces a novel neuroplastic process involving HMGB1-TLR4/RAGE neuroimmune signaling and REST-G9a epigenetic gene repression which results in reversible suppression of the BFCN phenotype that has broad implication for understanding how proinflammatory signaling impacts degeneration and brain diseases.

## Materials and methods

### In vivo AIE model

For in vivo AIE studies, male Wistar rats were bred at the University of North Carolina at Chapel Hill (UNC). Male subjects were used as we previously published comparable reductions of ChAT+ BFCNs in the adult basal forebrain of male and female rats [16, 25]. Subjects were housed in a temperature- (20 °C) and humidity-controlled vivarium on a 12 h/12 h light/dark cycle (light onset at 7:00 AM), and provided *ad libitum* access to food and water. This study was conducted in an AAALAC-accredited facility in strict accordance with NIH regulations for the care and use of animals in research. All experimental procedures reported in this study were approved by the Institutional Animal Care and Use Committee at UNC.

On postnatal day (P)21, rats were weaned and randomly assigned to either: (i) AIE or (ii) water control (CON) conditions. From P25 to P54, AIE subjects received a single daily intragastric (i.g.) administration of ethanol (EtOH; 5.0 g/kg, 20% EtOH, w/v) in the morning on a 2 day on/2 day off schedule whereas CON subjects received comparable volumes of water on an identical schedule as previously described by our laboratory [14, 15]. Tail blood was collected 1 h after treatment on P38 (AIE: 169 mg/dL [±20]) and P54 (AIE: 180 mg/dL [±22]) to assess blood ethanol concentration using a GM7 Analyzer (Analox; London, UK). All subjects evidenced dramatic body weight increases across age (main effect of Age: *p* = 0.000001, repeated measures ANOVA) that were unaffected by AIE treatment (main effect of Treatment: *p* = 0.41, repeated measures ANOVA). Subjects were sacrificed on P80 and brain tissue collected for immunohistochemistry (IHC; *n* = 10 subjects/group unless otherwise indicated) and real-time PCR (RTPCR; *n* = 6 [CON]; *n* = 7 [AIE] unless otherwise indicated).

For IHC experiments, animals were anesthetized with sodium pentobarbital (100 mg/kg, i.p.) and transcardially perfused with 0.1 M PBS followed by 4.0% paraformaldehyde. Brains were excised and post-fixed in 4.0% paraformaldehyde for 24 h at 4 °C followed by a 4 d fixation in 30% sucrose solution. Coronal sections were cut (40 µm) on a sliding microtome (MICROM HM450; ThermoScientific, Austin, TX, USA). For RTPCR experiments, animals were anesthetized with a lethal dose of sodium pentobarbital (100 mg/kg, i.p.) and transcardially perfused with 0.1 M PBS, brains excised, and basal forebrain tissue dissected and stored at −80 °C.

### Ex vivo organotypic FSC model

Ex vivo FSC studies were conducted using P8 male and female rat neonates (sex not tracked) from Charles River Laboratory (Wilmington, MA, USA) as previously described [13]. Two brain slices containing the basal forebrain (approximately Bregma: 1.00 mm – 0.20 mm based on the atlas of Paxinos and Watson [26]) from each animal were used for FSC. FSCs were treated with EtOH (100 mM) in media for 4 d (day ex vivo 12–16), unless otherwise indicated, in a dedicated humidified 5.0% CO_2_ incubator at 36.5 °C containing 1.0 L water saturated with equal concentrations of EtOH to balance EtOH evaporation. For the HMGB1 experiments, FSCs were treated with either disulfide HMGB1 (dsHMGB1; HMGBiotech, Milano, Italy, Cat. #HM-121; 0.1–100 ng/mL) or fully reduced HMGB1 (rHMGB1; HMGBiotech, Cat. #HM-115; 0.1–10 ng/mL). In some experiments, FSCs were treated with glycyrrhizin (HMGB1 inhibitor [Sigma-Aldrich, St. Louis, MO, Cat. #G2137; 100 µM]) [27], LPS-RS (TLR4 antagonist [InvivoGen, San Diego, CA, USA, Cat. #tlrl-prslps; 100 ng/mL]) [27], FPS-ZM1 (RAGE antagonist [Tocris, Bristol, UK, Cat. #945714-67-0; 10–1000 nM]), or UNC0642 (G9a inhibitor [Santa Cruz, Dallas, TX, USA, Cat. #sc397059; 1.0 µM]) [13] in combination with EtOH or HMGB1 as described in the Results section. For knockdown of REST, a Silencer Select REST siRNA cocktail and Silencer Select scrambled negative control siRNA (Ambion, Grand Island, NY, USA, Cat. #136124 [100 nM], #136125 [100 nM], #136126 [100 nM]; negative control siRNA, Cat. #4390843) was used based on the protocol for transfection as previously described [13]. For prevention studies, FSCs were transfected for 6 h prior to the addition of EtOH for 4 d. For the restoration studies, FSCs were treated with EtOH for 4 d, media changed and well inserts containing slices rinsed with fresh media, and transfected for 4 d. At the end of experimentation, slices were collected from tissue inserts for analysis. Wells containing dead or contaminated slices were immediately removed from experiments.

For IHC studies, membrane tissue inserts were collected from each well (*n* = 6 wells/group unless otherwise indicated), fixed in a solution of 4.0% paraformaldehyde and 5.0% sucrose in 0.1 M PBS (pH 7.4) for 24 h, and stored in 0.1 M PBS. For RNA (*n* = 6 wells/group) and DNA (*n* = 10 wells/group) extraction, slices were rinsed in cold 0.1 M PBS, removed from membrane tissue inserts, and stored at −80 °C.

### Assessment of neuronal cell death

Uptake of the fluorescent exclusion dye propidium iodide (PI) was used for determination of neuronal cell death as previously described [13, 28, 29]. Briefly, PI was added to the culture medium at the beginning of EtOH (100 mM) treatment at a concentration of 5.0 µg/mL and PI fluorescent images captured at 6 h, 24 h, and 96 h relative to time-matched CONs. PI intercalates into the DNA of non-viable cells but cannot enter viable cells as it is excluded by the plasma membrane providing a measure of cell death [29]. This method is well characterized for accurately measuring neuronal cell death in organotypic slice culture [13, 30]. PI fluorescent immunoreactive cells were imaged using AxioVision 3.1 software and quantified by an experimenter blind to condition using ImageJ^TM^ software.

### Post-mortem human basal forebrain tissue

Post-mortem human basal forebrain paraffin-embedded tissue samples from moderate drinking CON and AUD individuals (*n* = 10 subjects/group) were obtained from the New South Wales Brain Tissue Resource Centre (NSW BTRC [Ethnics Committee Approval Number: X11-0107]) at the University of Sydney (supported by the National Health and Medical Research Council of Australia-Schizophrenia Research Institute and the National Institute of Alcohol Abuse and Alcoholism [NIH (NIAAA) R24AA012725]). Subject information was collected from personal and next-of-kin interviews as well as medical records and is presented in Table 1. Only individuals with AUD uncomplicated by liver cirrhosis and/or nutritional deficiencies were included in the present study, and AUD diagnoses confirmed using the Diagnostic Instrument for Brain Studies that complies with the Diagnostic and Statistical Manual of Mental Disorders [31].

**Table 1:** Demographics of post-mortem human male moderate drinking control (CON) and alcohol use disorder (AUD) individuals.

Classification	Age of death	Brain weight (g)	PMI (h)	Brain pH	RIN	Clinical cause of death	Age of drinking onset	Lifetime alcohol consumption (kg)	Years drinking	BMI (kg/m^2^)
CON	53	1590	16	6.8	7.9	Cardiac	25	102	28	26
CON	48	1330	24	6.7	6.9	Cardiac	25	17	23	24
CON	44	1220	50	6.6	7.1	Cardiac	25	28	19	28
CON	60	1420	28	6.8	8	Cardiac	25	Unknown	35	29
CON	46	1320	29	6.1	4.4	Cardiac	25	115	21	Unknown
CON	24	1490	43	6.3	6.2	Cardiac	20	15	4	38
CON	50	1426	30	6.4	7.5	Cardiac	25	Unknown	Unknown	28
CON	62	1430	46	7	8.8	Cardiac	25	5	*7*	*33*
CON	50	1596	40	6.9	8.6	Cardiac	25	18	25	29
CON	40	1441	27	6.8	7.4	Cardiac	25	47	16	35
AUD	25	1400	43.5	6.7	6.9	Toxicity	16	552	9	19
AUD	50	1520	17	6.3	7	Cardiac	18	2453	32	24
AUD	44	1360	15	6.5	7.9	Cardiac	20	639	10	24
AUD	42	1400	41	6.5	8	Toxicity	18	1472	24	24
AUD	45	1580	18.5	6.6	7.9	Respiratory	15	1800	29	29
AUD	61	1588	59	6.6	6.1	Cardiac	16	8052	43	25
AUD	49	1600	44	6.4	6.4	Cardiac	16	1012	33	26
AUD	49	1420	16	6.2	6.2	Cardiac	14	613	35	33
AUD	61	1340	23.5	6.9	8.3	Cardiac	17	5621	44	25
AUD	50	1470	34.5	6.9	7.3	Respiratory	16	5212	34	28

Age of drinking onset is significantly different (t(18) = −10.7, *p* = 0.0001) between CON (24.5 ± 0.5) and AUD individuals (16.6 ± 0.5). Lifetime alcohol consumption (CON: 43 kg ±15, AUD: 2743 kg ±829; t(9.0) = 3.3, *p* = 0.010, Welch’s *t* test) and body mass index (BMI; CON: 30 kg/m^2^ ± 1, AUD: 26 kg/m^2^ ± 1; t(17) = −2.3, *p* = 0.035) are significantly different between groups. No differences were observed regarding age of death (*p* = 0.983), brain weight (*p* = 0.401), post-mortem interval (PMI; *p* = 0.728), brain pH (*p* = 0.502), RNA integrity number (RIN; *p* = 0.869), or years drinking (*p* = 0.077).

### Immunohistochemistry

Free-floating basal forebrain in vivo (every 6^th^ section; approximately Bregma: 1.00 mm – 0.20 mm based on the atlas of Paxinos and Watson [26]) and ex vivo immunohistochemistry experiments were conducted as previously described [13–15, 32]. Sections were incubated in a primary antibody solution containing blocking solution with either goat anti-ChAT (Millipore, Temecula, CA, USA, Cat. #AB144P), rabbit anti-parvalbumin (Abcam, Cambridge, MA, USA, Cat. #ab11427), mouse anti-NeuN (Millipore, Cat. #MAB377), or rabbit anti-REST (Abcam, Cat. #ab202962). Negative control for non-specific binding was conducted on in vivo sections employing the above-mentioned procedures, omitting the primary antibody.

Immunohistochemical assessment of paraffin-embedded post-mortem human basal forebrain sections were conducted as previously described [12, 32, 33]. Slides were incubated in a primary antibody solution containing Dako antibody diluent (Dako North America, Carpinteria, CA, Cat. #S0809) and rabbit anti-REST (Bioss Antibodies, Woburn, MA, Cat. #bs-2590R).

### Microscopic quantification and image analysis

Across experiments, BioQuant Nova Advanced Image Analysis software (R&M Biometric, Nashville, TN, USA) was used for image capture and quantification of IHC. Representative images were captured using an Olympus BX50 microscope and Sony DXC-390 video camera linked to a computer. For each measure, the microscope, camera, and software were background-corrected and normalized to preset light levels to ensure fidelity of data acquisition. A modified unbiased stereological quantification method was used, which was performed by experimenters blind to treatment, to quantify ChAT+IR, parvalbumin+IR, NeuN+IR, and REST+IR cells in the basal forebrain. The anterior commissure was used as a landmark to assist with identification of the basal forebrain. We previously reported that comparison of traditional unbiased stereological methodology with our modified unbiased stereological approach yielded nearly identical values for heterogeneously distributed cell populations [34]. The outlined regions of interest were determined and data expressed as cells/mm^2^. ChAT+IR somal size was assessed using BioQuant Nova Advanced Image Analysis software (R&M Biometric).

### Fluorescent immunohistochemistry and microscopy

Paraffin-embedded human basal forebrain sections were deparaffinized, washed in PBS, and antigen retrieval performed by incubation in Citra solution (BioGenex) for 1 h at 70 °C. Following incubation in blocking solution (MP Biomedicals), slides were incubated for 48 h at 4 °C in a primary antibody solution consisting of Dako antibody diluent (Dako North America) with rabbit anti-REST (Bioss Antibodies) and goat anti-ChAT (Millipore). Slides were washed in PBS and incubated for 1 h at room temperature with Alexa Fluor 488 (Invitrogen, Cat. #A32814) and Alexa Fluor 594 (Invitrogen, Cat. #A21207) secondary antibodies. Secondary-only negative controls were performed without primary antibody incubation. Immunofluorescent images were obtained using a Nikon DS-Ri2 scope (Nikon Inc., Melville, NY) and colocalization quantified using NIS Elements AR46 analysis software (Nikon Inc.).

### ELISA

At the conclusion of FSC studies, media was collected and used for detection of released HMGB1. HMGB1 ELISA was performed according to the manufacturer’s protocol (IBL International, Hamburg, Germany, Cat. #ST51011).

### RNA extraction and RTPCR

Across experiments, total mRNA was extracted and reverse transcribed from in vivo basal forebrain and ex vivo FSC samples as previously described [16, 35, 36]. RTPCR reactions were run on a BioRad CFX system (BioRad, Hercules, CA, USA) and primer sequences are presented in Table 2. Differences in primer extension of genes of interest between groups are expressed as cycle time (Ct) values normalized to a housekeeping gene (i.e., *β actin* or *18S*), and relative differences between groups calculated using the ΔΔCt method and expressed as the percent difference relative to CONs.

**Table 2:** List of primer sequences for RTPCR analysis.

Primer	Forward	Reverse
*Ache*	GACTGCCTTTATCTTAATGTG	CGGCTGATGAGAGATTCATTG
*Acly*	CAGCAGGACAGCGTCTTTTTC	GGGATCTTGGACTTGGGACT
*Ccl2*	CTGGGCCTGTTGTTCACAGTTGC	CTACAGAAGTGCTTGAGGTGGTTG
*Cd11b*	CTGGTACATCGAGACTTCTC	TTGGTCTCTGTCTGAGCCTT
*Chat*	GCCCAACCAAGCCAAGCAAT	AAATGTCTTTGCGGGTGCCG
*Chrm2*	CCATCAATCCGGCCTGCTAT	TCGCTGTTCTTCCCCAAGAC
*Chrna6*	GTCGATGCCTCTGGCTACAA	CACCACAATGGACAGCGTGA
*Chrna7*	TTCTCCTCTATAACAGTGCTGATG	GACCACCCTCCATAGGACCA
*Chrnb2*	GACCACATGCGAAGTGAGGA	AAGATCCACAGGAACAGGCG
*Cht*	CATCACAGAACCTCACTCACAC	GCAAGAGGCTGAAACATTTGGG
*G9a*	CTCCGGTCCCTTGTCTCC	CTATGAGAGGTGTCCCCCAA
*Gfap*	AATGACTATCGCCGCCAACT	CGAGTGCCTCCTGGTAACTC
*Hmgb1*	ATGGGCAAAGGAGATCCTA	ATTCATCATCATCATCTTCT
*Iba1*	GGCAATGGAGATATCGATA	AGAATCATTCTCAAGATGGC
*Il1β*	GAAACAGCAATGGTCGGGAC	AAGACACGGGTTCCATGGTG
*Il6*	CTGGTCTTCTGGAGTTCCGTT	GGTCTTGGTCCTTAGCCACTC
*Lhx8*	GCCTTGGTAGAGGAGAAGGTC	TGGCTGGCTTTGGATGATTGA
*Neun*	CCCACCACTCTCTTGTCCGT	GGGCCGATGGTATGATGGTAG
*Ngfr*	GCTGCTGATTCTAGGGATGTC	CAGTCTCCTCGTCCTGGTAGT
*Rage*	CAGTAGGAAGTGGGGCAGAC	ATGGCACAGGTCAGGGTCAC
*Rest*	ACTACACGGCACACCTGAAG	GAGGTTTAGGCCCGTTGTGA
*Tnfα*	ATGTGGAACTGGCAGAGGAG	ACGAGCAGGAATGAGAAGAAG
*Tlr4*	CCAGAGCCGTTGGTGTATCT	TCAAGGCTTTTCCATCCAAC
*Trka*	CCATATCAAGCGCCAGGACA	GCAGTTTTGCATCAGGTCCG
*Vacht*	AGGCCACATCGTTCACTCTC	GGCGGTTCATCAAGCAACAC
*β actin*	CTACAATGAGCTGCGTGTGGC	CAGGTCCAGACGCAGGATGGC
*18s*	CGGGGAATCAGGGTTCGATT	TCGGGAGTGGGTAATTTGCG

### Chromatin immunoprecipitation (ChIP)

ChIP was performed as previously described by our laboratory [13–15]. Sheared chromatin was incubated overnight at 4 °C with validated ChIP-grade antibodies against H3K9me2 (Abcam, Cat. #ab1220) or REST (Millipore, Cat. #17-641). The resulting DNA was quantified using qPCR with SSOAdvanced Universal SYBR Green Supermix (Life Technologies, Cat. #4367659) and primers for promoter and promoter CpG islands at *Chat*, *Trka*, *Lhx8*, and *Parvb* genes (Table 3). The ΔΔCt method was used to determine fold occupancy relative to CONs and was normalized to the Input DNA fraction.

**Table 3:** List of primer sequences for ChIP analysis.

Primer	Forward	Reverse	Target
*Chat* Promoter	ACTTGATTGCTGCCTCTCTC	GGGATGGTGGAAGATACAGAAG	Promoter
*Chat* CpG Promoter	TGCATCTGGAGCTCAAATCGT	GGGGATAGTGGTGACGTTGT	Promoter CpG island
*Trka* Promoter	CCTCACCGTGCACTTTACCT	AGGGTCTGGAGAGCGTACAT	Promoter
*Trka* CpG Promoter	TCAAGCAAGGCTCCGAACAG	CACAGGGTGGCGCTAGAAG	Promoter CpG island
*Lhx8* Promoter	ATCGGAGGCGGTGTATGTTC	TGGGCCTGGTTCGGATTAAG	Promoter
*Parvb* Promoter	CAGAAGATGGTCCCTGACGG	ACCTTCAAGCTGAACGGGTC	Promoter

### Statistical analysis

Statistical analysis was performed using GraphPad Prism 8 (San Diego, CA). Two-tailed Student’s *t* tests were used to assess BECs, RTPCR, IHC, and ChIP data. Levene’s test for equality of variance was performed for each analysis. When reported in the Results, Welch’s *t* tests were used to assess data with unequal variances. All time course and dose response data was assessed using a one-way ANOVA with follow-up Dunnett’s multiple comparison tests or Sidak correction for multiple comparisons. Body weight data was assessed using repeated measures ANOVA. Pharmacological studies were analyzed using 2 × 2 ANOVAs with Tukey’s HSD post-hoc analyses when appropriate. If significant interactions were not observed, follow-up *t* tests were performed to determine pharmacological blockade. Two-tailed Pearson’s r was used for correlative analyses. Sample size was based on prior in vivo and ex vivo studies. Sample size estimates were not used in the present study. All values are reported as mean ± SEM.

## Results

### In vivo AIE and ex vivo EtOH decrease expression of cholinergic phenotype markers in the basal forebrain

**Fig. 1:**
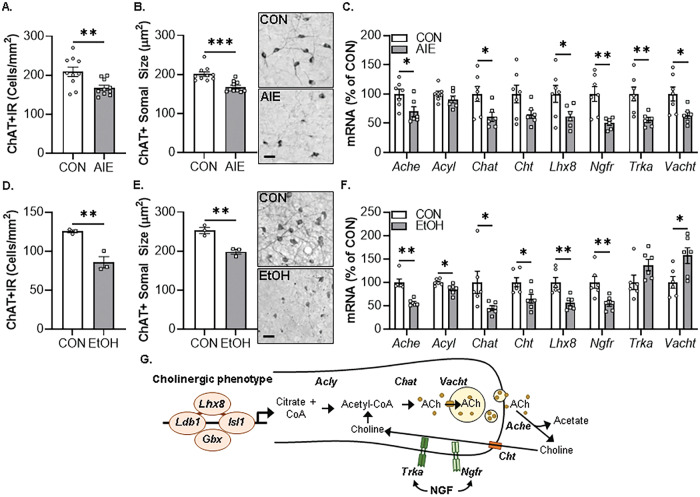
In vivo AIE- and ex vivo EtOH-induced reductions of ChAT+IR forebrain cholinergic neurons (BFCNs) and cholinergic phenotype gene expression in the basal forebrain. **A** Modified unbiased stereological assessment revealed that in vivo AIE treatment (5.0 g/kg EtOH, i.p. from postnatal day [P]25 to P54) caused a 20% (±3%) reduction of ChAT+IR BFCNs in the adult (P80) basal forebrain relative to CONs (*t*[18] = 3.0, *p* = 0.008, Student’s *t* test; *n* = 10 subjects/group). **B** Assessment of BFCN somal size revealed an in vivo AIE-induced 17% (±2%) reduction in somal size of the remaining ChAT+ neurons relative to CONs (*t*[18] = 4.6, *p* = 0.0002, Student’s *t* test; *n* = 10 subjects/group). Representative photomicrographs of ChAT+IR BFCNs in CON- and AIE-treated subjects. Note the somal shrinkage of the remaining ChAT+ BFCNs following AIE treatment relative to CONs. **C** Real-time PCR (RTPCR) analysis revealed that in vivo AIE treatment decreased mRNA expression of *Ache* (30% [±9%]; *t*[11] = 2.6, *p* = 0.026, Student’s *t* test), *Chat* (39% [±8%]; *t*[11] = 2.5, *p* = 0.029, Student’s *t* test), *Ngfr* (50% [±4%]; *t*[7.2] = 3.8, *p* = 0.006, Welch’s *t* test), *Trka* (45% [±5%]; *t*[11] = 3.3, *p* = 0.007, Student’s *t* test), and *Vacht* (36% [±6%]; *t*[11] = 2.6, *p* = 0.027, Student’s *t* test) as well as the cholinergic lineage transcription factor *Lhx8* (38% [±8%]; *t*[11] = 2.2, *p* = 0.050, Student’s *t* test) in the adult (P80) basal forebrain relative to CONs (*n* = 6-7 subjects/group). **D** Modified unbiased stereological assessment revealed that ex vivo EtOH treatment (100 mM EtOH for 96 h) of basal forebrain slice culture (FSC) caused a 32% (±6%) reduction of ChAT+IR BFCNs relative to CONs (*t*[4] = 5.3, *p* = 0.006, Student’s *t* test; *n* = 3 wells/group). **E** Assessment of BFCN somal size revealed an ex vivo EtOH-induced 22% (±2%) reduction in somal size of the remaining ChAT+ neurons relative to CONs (*t*[4] = 5.6, *p* = 0.005, Student’s *t* test; *n* = 3 wells/group). Representative photomicrographs of ChAT+IR BFCNs in CON- and EtOH-treated FSCs. Note the somal shrinkage of the remaining ChAT+ BFCNs following EtOH treatment relative to CONs. **F** RTPCR analysis revealed that ex vivo EtOH treatment (100 mM EtOH for 96 h) of FSC decreased mRNA expression of *Ache* (43% [±3%]; *t*[10] = 5.6, *p* = 0.0002, Student’s *t* test), *Acyl* (15% [±4%]; *t*[10] = 2.9, *p* = 0.016, Student’s *t* test), *Chat* (55% [±5%]; *t*[10] = 2.3, *p* = 0.045, Student’s *t* test), *Cht* (35% [±10%]; *t*[10] = 2.5, *p* = 0.030, Student’s *t* test), and *Ngfr* (45% [±6%]; *t*[10] = 3.2, *p* = 0.009, Student’s *t* test) as well as the cholinergic lineage transcription factor *Lhx8* (44% [±6%]; *t*[10] = 3.7, *p* = 0.007, Student’s *t* test) relative to CONs (Student’s *t* test; *n* = 6 wells/group). **G** Schematic depicting cholinergic phenotype and lineage genes. Ache acetylcholinesterase, Acly ATP citrate lyase, Chat choline acetyltransferase, Cht high-affinity choline transporter, Gbx gastrulation brain homeobox, Isl1 insulin gene enhancer protein 1, Ldb1 LIM domain-binding protein 1, Lhx8 LIM/homeobox protein 8, Ngfr nerve growth factor receptor, Trka tropomyosin receptor kinase A, Vacht vesicular acetylcholine transporter. RTPCR analyses were run in duplicate. Scale bar = 50 μm. Data are presented as mean ± SEM. *p ≤ 0.05, **p < 0.01, ***p < 0.001.

In vivo AIE treatment induces lasting HMGB1-TLR4/RAGE neuroimmune signaling in the adult basal forebrain linked to persistent, but reversible, epigenetic repression of the adult BFCN phenotype [8, 14–16]. In the present series of experiments, we replicate and extend these discoveries using an ex vivo organotypic FSC model to investigate the molecular mechanisms of EtOH-induced epigenetic repression of the BFCN phenotype. We first evaluated the effect of EtOH on ChAT+IR loss and somal shrinkage of the residual ChAT+ neurons in our ex vivo FSC model by comparison to our in vivo AIE model. Administration of AIE caused a 20% (±3%) reduction of adult (P80) ChAT+IR BFCNs (Fig. 1A), consistent with previously published studies [8, 15, 16]. Further, we report a 17% (±2%) reduction in somal size of the remaining ChAT+ BFCNs in the adult basal forebrain following AIE (Fig. 1B). In the FSC model, EtOH caused a 32% (±6%) reduction of ChAT+IR BFCNs (Fig. 1D) that was accompanied by a 22% (±2%) reduction in somal size of the remaining ChAT+ neurons (Fig. 1E). While somal shrinkage and loss of ChAT+ BFCNs could be interpreted as cell death and/or autophagy, no changes in expression of the neuronal gene *Neun* were found across models (Table 4) consistent with prior studies reporting no changes in NeuN+IR neurons in the basal forebrain following AIE treatment [14, 15]. In the FSC model, no change in NeuN+IR neurons was found following application of EtOH (50 mM and 100 mM; Supplementary Fig. 1A) or the cell death marker PI following EtOH application (100 mM) across a 6 h to 96 h time course (Supplementary Fig. 1B). The FSC model was used to assess EtOH concentration (10, 30, 50, and 100 mM for 96 h) and time course (24, 48, and 96 h) effects on ChAT+IR BFCNs. Application of EtOH at a concentration of 50 mM and 100 mM, but not 10 mM or 30 mM, significantly decreased ChAT+IR BFCNs (Supplementary Fig. 2A). Time course analysis revealed that EtOH at a concentration of 100 mM significantly decreased ChAT+IR BFCNs at 24 h, 48 h, and 96 h relative to CONs assessed at 96 h (Supplementary Fig. 2B). The effect of EtOH appear specific to ChAT+IR BFCNs as parvalbumin+IR GABAergic neuron populations were unaffected in either the in vivo AIE or ex vivo FSC model (Supplementary Fig. 3A/B). While it is difficult to relate ex vivo slice culture to the in vivo AIE model, our findings indicate that the FSC model recapitulates the in vivo AIE-induced ChAT+ somal shrinkage and reduction of ChAT+IR BFCNs.

**Table 4:** Effect of in vivo AIE and ex vivo EtOH on expression of cholinergic receptor, cellular, and neuroimmune genes in the basal forebrain.

		In vivo AIE model		Ex vivo FSC model	
		CON	AIE	CON	EtOH
Cholinergic receptor genes	*Chrnb2*	100 ± 4	94 ± 5	**100** ± **7**	**55** ± 6**
	*Chrna6*	100 ± 17	113 ± 41	100 ± 76	455 ± 195
	*Chrna7*	**100** ± **10**	**65** ± 6*	**100** ± **8**	**39** ± 4**
	*Chrm2*	100 ± 11	87 ± 5	**100** ± **8**	**64** ± 11*
Cellular genes	*Cd11b*	100 ± 12	108 ± 6	100 ± 9	82 ± 18
	*Iba1*	100 ± 9	118 ± 7	100 ± 8	83 ± 16
	*Gfap*	100 ± 24	86 ± 5	100 ± 11	73 ± 13
	*Neun*	100 ± 19	110 ± 6	100 ± 25	86 ± 26
Neuroimmune genes	*Tnfα*	100 ± 10	89 ± 9	100 ± 6	90 ± 21
	*Il1β*	**100** ± **12**	**142** ± 15*	**100** ± **10**	**435** ± 93*
	*Il6*	100 ± 17	77 ± 14	**100** ± **12**	**257** ± 19**
	*Ccl2*	**100** ± **21**	**203** ± 32*	**100** ± **11**	**231** ± 51*

Bold indicates signficant changes. *p < 0.05, **p < 0.01.

Cholinergic neurons are defined by expression of the ACh-synthesizing enzyme ChAT as well as numerous molecules involved in the synthesis and packaging of ACh into synaptic vesicles for release and key cholinergic trophic factor receptors for NGF (Fig. 1G). Assessment of cholinergic phenotype genes in the AIE model reveal decreased expression of multiple cholinergic transcriptome genes, including *Ache* (30% [±9%]), *Chat* (39% [±8%]), *Ngfr* (50% [±4%]), *Trka* (45% [±5%]), and *Vacht* (36% [±6%]) as well as the cholinergic lineage transcription factor *Lhx8* (38% [±8%]) (Fig. 1C). This was accompanied by reduced mRNA expression of the cholinergic receptor gene *Chrna7* (Table 4). In our FSC model, ex vivo application of EtOH decreased expression of *Ache* (43% [±3%]), *Acyl* (15% [±4%]), *Chat* (55% [±5%]), *Cht* (35% [±10%]), and *Ngfr* (45% [±6%]) as well as the cholinergic lineage transcription factor *Lhx8* (44% [±6%]) (Fig. 1F). The observed reduction and somal shrinkage of ChAT+IR BFCNs in the ex vivo model was accompanied by reduced mRNA expression of the cholinergic receptor gene *Chrna7* as well as *Chrnb2* and *Chrm2* (Table 4). Together, these data reveal comparable in vivo and ex vivo EtOH-induced reductions of ChAT+IR BFCNs, somal shrinkage of the remaining ChAT+ neurons, and decreased expression of cholinergic phenotype and lineage genes in the basal forebrain.

### EtOH-induced HMGB1 and neuroimmune signaling in the basal forebrain contributes to the loss of cholinergic phenotype

**Fig. 2:**
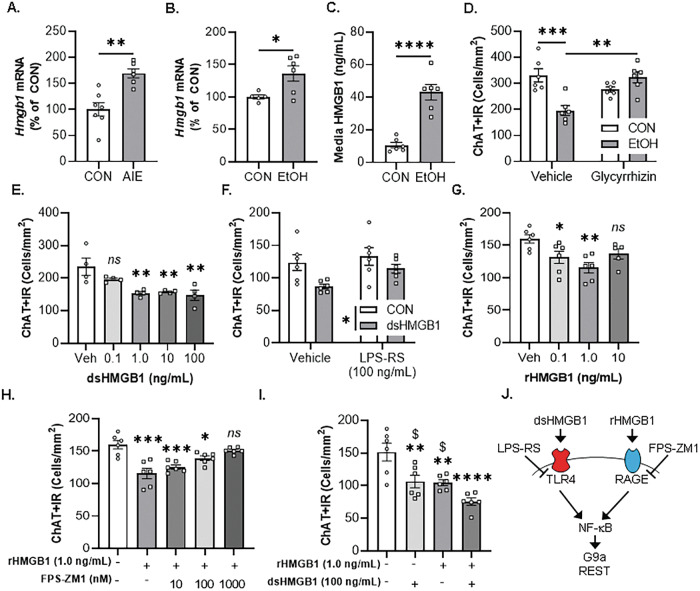
In vivo AIE and ex vivo EtOH increase expression of HMGB1, contributing to loss of ChAT+IR basal forebrain cholinergic neurons (BFCNs). **A** Real-time PCR (RTPCR) analysis revealed in vivo AIE treatment (5.0 g/kg EtOH, i.p. from postnatal day [P]25 to P54) increased *Hmgb1* mRNA approximately 1.7-fold in the adult (P80) basal forebrain relative to CONs (*t*[11] = 4.3, *p* = 0.001, Student’s *t* test; *n* = 6-7 subjects/group). **B** RTPCR analysis revealed that direct application of EtOH (100 mM; 96 h) to basal forebrain slice culture (FSC) media increased *Hmgb1* mRNA approximately 1.4-fold (*t*[5.7] = 2.9, *p* = 0.028, Welch’s *t* test) relative to CONs. **C** ELISA revealed EtOH treatment caused an approximate 4.1-fold increase in media levels of HMGB1 in the FSC relative to CONs (*t*[10] = 6.5, *p* = 0.00007, Student’s *t* test; *n* = 6 wells/group). **D** Modified unbiased stereological assessment revealed that direct application of EtOH to FSC media caused a 41% (±6%) reduction of ChAT+IR BFCNs relative to CONs (*p* = 0.0006, Tukey’s HSD). Treatment with the HMGB1 inhibitor glycyrrhizin (100 μM; 96 h) did not affect ChAT+ expression in CON FSCs, but blocked the EtOH-induced loss of ChAT+IR BFCNs (*p* = 0.001, Tukey’s HSD; *n* = 6 wells/group) relative to Veh/EtOH (interaction of 2 × 2 ANOVA: *F*[1,20] = 20.9, *p* = 0.0002). **E** Modified unbiased stereological assessment revealed that direct application of dsHMGB1 to FSC media for 24 h (*F*[4,15] = 6.9, *p* = 0.0023, one-way ANOVA) caused a dose-dependent (0.1 ng/mL: *p* = 0.195, 1.0 ng/mL: *p* = 0.004, 10 ng/mL: *p* = 0.005, 100 ng/mL: *p* = 0.002, Dunnett’s test) reduction of ChAT+IR BFCNs (*n* = 4 wells/group). **F** Modified unbiased stereological assessment revealed that direct application of dsHMGB1 (100 ng/mL; 24 h) to FSC media significantly reduced ChAT+IR BFCNs (Main effect of dsHMGB1: *F*[1,20] = 7.9, *p* = 0.011) relative to CONs. Treatment with the TLR4 antagonist LPS-RS (100 ng/mL; 24 h) led to an insignificant trend toward increased ChAT+IR BFCNs relative to vehicle-treated FSCs (Main effect of LPS-RS: *F*[1,20] = 3.5, *p* = 0.077, 2 × 2 ANOVA; *n* = 6 wells/group). **G** Modified unbiased stereological assessment revealed that direct application of rHMGB1 to FSC media for 24 h (*F*[3,19] = 5.4, *p* = 0.007, one-way ANOVA) caused a dose-dependent (0.1 ng/mL: *p* = 0.050, 1.0 ng/mL: *p* = 0.002, 10 ng/mL: *p* = 0.148, Dunnett’s test) reduction of ChAT+IR BFCNs (*n* = 5-6 wells/group). **H** Modified unbiased stereological assessment revealed that direct application of rHMGB1 (1.0 ng/mL; 24 h) to FSC media (*F*[4,25] = 12.2, *p* = 0.0001, one-way ANOVA) reduced expression of ChAT+IR BFCNs (*p* = 0.0001, Dunnett’s test), an effect that was blocked by FPS-ZM1 at a concentration of 1000 nM (*p* = 0.565, Sidak test) relative to CONs (*n* = 6 wells/group). **I** Modified unbiased stereological assessment revealed that direct application of dsHMGB1 (100 ng/mL; 24 h, *p* = 0.006, Dunnett’s test) and rHMGB1(1.0 ng/mL; 24 h, *p* = 0.005, Dunnett’s test) individually decreased ChAT+IR BFCNs whereas combined dsHMGB1+rHMGB1 caused a greater reduction of ChAT+IR BFCNs than either HMGB1 redox form alone (dsHMGB1: *p* = 0.013, rHMGB1: *p* = 0.019, Dunnett’s test; *n* = 6 wells/group). **J** Simplified schematic depicting dsHMGB1 binding to TLR4 and rHMGB1 binding to RAGE, which signal through the nuclear transcription factor NF-κB that are expressed by BFCNs [14], to induce the epigenetic repressive methyltransferase G9a and REST [49–51]. Depicted is the TLR4 antagonist LPS-RS and the RAGE antagonist FPS-ZM1. RTPCR analyses were run in duplicate. Data are presented as mean ± SEM. *p ≤ 0.05, **p < 0.01, ***p < 0.001, ****p < 0.0001, relative to CON/Veh condition; ^$^*p* < 0.05, relative to dsHMGB1+rHMGB1 condition.

Previous studies report AIE causes lasting increases of HMGB1, TLR4, RAGE, and other neuroimmune signaling molecules in brain, including the basal forebrain, in association with neuropathology [10, 14–16, 36, 37]. Analysis of *Hmgb1* mRNA in the adult basal forebrain revealed an AIE-induced approximate 1.7-fold increase (Fig. 2A), consistent with previous studies [14]. Induction of *Hmgb1* mRNA in the adult basal forebrain was accompanied by increased expression of the proinflammatory cytokine *Il1β* and the chemokine *Ccl2* (Table 4). In the FSC model, EtOH increased *Hmgb1* mRNA 1.4-fold (Fig. 2B) and increased HMGB1 media levels approximately 4.1-fold (Fig. 2C). The increase of HMGB1 media levels in the FSC model was accompanied by increased mRNA expression of the proinflammatory cytokines *Il1β* and *Il6* as well as the chemokine *Ccl2* (Table 4). We did not observe changes in microglial genes *Iba1* or *Cdllb*, the astrocyte gene *Gfap*, or the neuronal gene *Neun* in either model (Table 4). These findings are consistent with EtOH-induced release of HMGB1 and proinflammatory gene induction contributing to the loss of ChAT+IR BFCNs, but not neuronal death. We next determined whether HMGB1 contributes to the EtOH-induced loss of ChAT+IR BFCNs by applying the direct HMGB1 inhibitor glycyrrhizin [38] to FSC media during EtOH treatment. Consistent with our earlier findings, EtOH caused a 41% (±6%) reduction of ChAT+IR BFCNs, an effect that was blocked in EtOH-treated FSCs by glycyrrhizin (Fig. 2D). These data support EtOH-induced HMGB1 neuroimmune signaling, perhaps in ChAT+IR neurons, in the loss of ChAT+IR BFCNs.

TLR4 and RAGE are the primary neuroimmune receptors for HMGB1 mediation of proinflammatory signaling induction through NF-κB [39–44]. HMGB1 contains three cysteine residues (i.e., C23, C45, and C106) that are modifiable by redox reactions to produce multiple HMGB1 redox isoforms with differing extracellular activities. The redox state of HMGB1 dictates receptor binding as dsHMGB1 containing C23 and C45 disulfide linkage and C106 in a reduced form as a thiol, binds to TLR4 [45] whereas rHMGB1, in which all three cysteine residues are reduced, binds to RAGE [46, 47]. Disulfide HMGB1 facilitates TLR4-dependent signaling with high affinity (apparent *K*_d_ = 12 nM) whereas reduced HMGB1 had an approximate 1000-fold reduction in TLR4-dependent signaling [45, 48]. Our AIE studies find colocalization of TLR4+, RAGE+, and NF-κBp65+IR with ChAT+IR BFCNs [14], consistent with EtOH exposure increasing HMGB1-TLR4/RAGE signaling within ChAT+ neurons. Using our FSC model, we investigated direct HMGB1 activation in the loss of ChAT+IR BFCNs. To investigate TLR4 involvement, we applied dsHMGB1 and found dose-dependent reductions of ChAT+IR BFCNs at concentrations of 1.0 ng/mL (35% [±2%]), 10 ng/mL (33% [±1]), and 100 ng/mL (37% [±7%]) (Fig. 2E). Our observation that higher concentrations of dsHMGB1 did not lead to further reductions of ChAT+IR neurons is consistent with previous in vivo AIE studies reporting that the loss of BFCNs is limited to approximately 25–30% of the population, with somal shrinkage of the remaining ChAT+IR neurons. We next determined whether the TLR4 antagonist LPS-RS (100 ng/mL; 24 h) [13] blocked the dsHMGB1-induced loss of ChAT+IR BFCNs. Consistent with our findings above, dsHMGB1 (100 ng/mL) led to an overall reduction of ChAT+IR BFCNs whereas treatment with LPS-RS led to an insignificant trend toward increased ChAT+IR neurons (Fig. 2F). To determine RAGE involvement, we next applied rHMGB1 to the FSC model and report a dose-dependent reduction of ChAT+IR BFCNs at concentrations of 0.1 ng/mL (18% [±6%]) and 1.0 ng/mL (28% [±5%]) (Fig. 2G). To confirm rHMGB1-RAGE signaling, we assessed if the RAGE antagonist FPS-ZM1 blocked the rHMGB1-induced loss of ChAT+IR BFCNs. rHMGB1 (1.0 ng/mL; 24 h) caused a 28% (±4%) reduction of ChAT+IR BFCNs, an effect that was blocked by FPS-ZM1 treatment (1000 nM; Fig. 2H). We next determined if application of both dsHMGB1 and rHMGB1 would additively decrease ChAT+IR BFCNs in the FSC model. Independently, dsHMGB1 and rHMGB1 decreased ChAT+IR BFCNs consistent with the above studies; surprisingly, combined dsHMGB1 and rHMGB1 caused a greater reduction of ChAT+IR BFCNs (i.e., ~40% reduction) than either HMGB1 redox form alone (Fig. 2I). These findings suggest that multiple forms of HMGB1, signaling through TLR4 and/or RAGE, are able to reduce ChAT+IR BFCNs. Together with our in vivo data, these findings support the hypothesis that EtOH-induced release of HMGB1 binds to TLR4 and RAGE receptors leading to nuclear translocation of NF-κBp65 culminating in loss of the cholinergic phenotype through activation of repressive epigenetic signaling mechanisms [49–51] (Fig. 2J).

### EtOH-induced REST/G9a in the basal forebrain contributes to epigenetic repression of BFCN phenotype genes

**Fig. 3:**
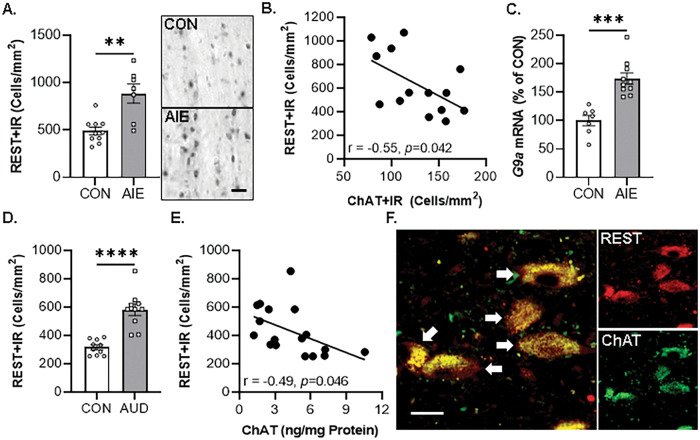
Increased expression of epigenetic repressive molecules in the basal forebrain of adult AIE-treated rats and post-mortem human individuals with AUD. **A** Modified unbiased stereological assessment revealed that in vivo AIE treatment (5.0 g/kg EtOH, i.p. from postnatal day [P]25 to P54) caused an approximate 1.8-fold increase in REST+IR in the adult (P80) basal forebrain relative to CONs (*t*[8] = 3.6, *p* = 0.007, Welch’s *t* test; *n* = 7–10 subjects/group). Representative photomicrographs of REST+IR in the basal forebrain of CON- and AIE-treated subjects. Scale bar = 50 μm. **B** Immunohistological expression of REST+IR negatively correlates with expression of ChAT+IR BFCNs (*r* = −0.55, *N* = 14, *p* = 0.042, Pearson’s r) in the adult (P80) basal forebrain. **C** Real-time PCR (RTPCR) analysis revealed in vivo AIE treatment increased *G9a* mRNA approximately 1.7-fold in the adult basal forebrain relative to CONs (*t*[15] = 5.2, *p* = 0.0001, Student’s *t* test; *n* = 7–10 subjects/group). **D** Modified unbiased stereological assessment revealed an approximate 2.0-fold increase of REST+IR in post-mortem human basal forebrain of individuals with AUD relative to age-matched moderate drinking CONs (*t*[11.9] = 6.0, *p* = 0.0001, Welch’s *t* test; *n* = 10 individuals/group). **E** Immunohistological expression of REST+IR negatively correlates with protein expression of ChAT from a previously published study comprising the same cohort [8] (Pearson’s *r* = −0.49, *N* = 17, *p* = 0.046). **F** Immunofluorescent co-labeling revealed a high degree of REST (red) colocalization with ChAT+IR BFCNs in the post-mortem human basal forebrain. White arrows indicate REST+IR cells that colocalize with ChAT (yellow). Scale bar = 20 μm. **p < 0.01, ***p < 0.001, ****p < 0.0001.

In vivo AIE increases occupancy of the epigenetic repressive marker H3K9me2 at cholinergic phenotype genes (i.e., *Chat* and *Trka*) that parallels loss of ChAT+IR BFCNs, which are both reversed by post-AIE adult exercise and galantamine treatment [14, 15]. The histone methyltransferase G9a, which is recruited by the repressive transcription factor REST, reversibly suppresses gene transcription through dimethylation of H3K9 [52, 53]. In vivo, we report AIE caused an approximate 1.8-fold increase of REST+IR in the basal forebrain (Fig. 3A), which was negatively correlated with ChAT+IR expression (Fig. 3B). Further, AIE increased *G9a* mRNA expression approximately 1.7-fold in the basal forebrain (Fig. 3C). Similarly, REST+IR is increased approximately 2.0-fold in the post-mortem human basal forebrain of individuals with AUD (Fig. 3D). Expression of REST+IR negatively correlated with ChAT protein expression [8] (Fig. 3E), consistent with our observation of REST colocalization with ChAT+IR BFCNs (Fig. 3F).

**Fig. 4:**
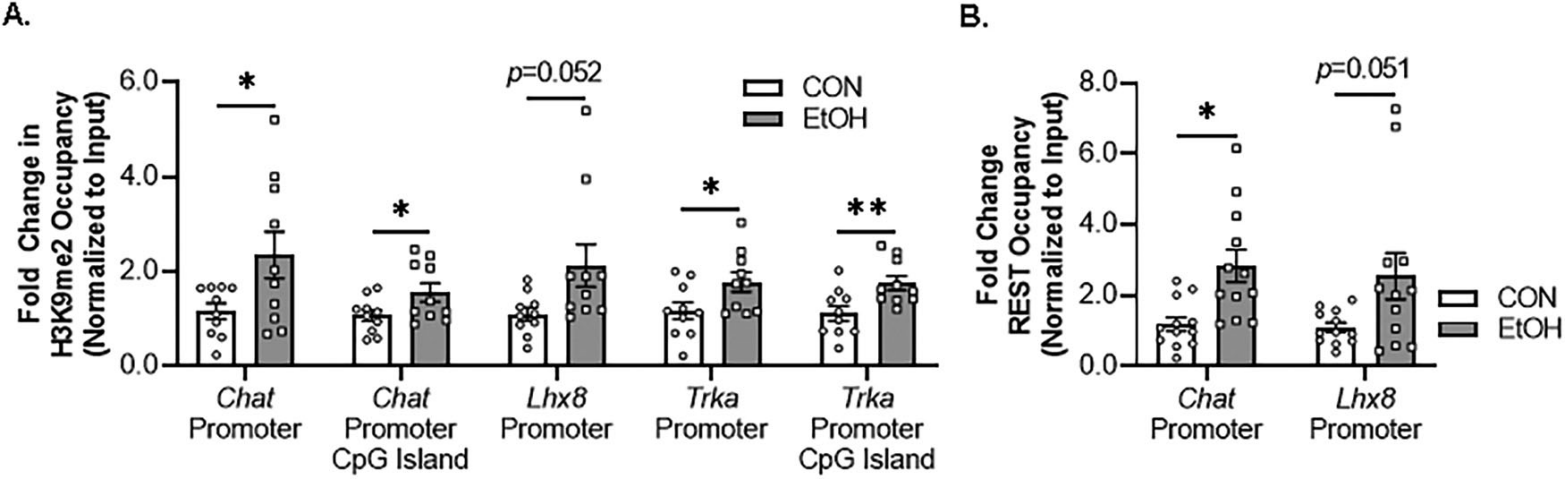
Direct EtOH treatment ex vivo increases histone methylation and RE1 silencing transcription factor (REST) occupancy at cholinergic gene promoters. **A** Chromatin immunoprecipitation (ChIP) revealed that direct application of EtOH (100 mM; 96 h) to basal forebrain slice culture (FSC) media increased histone 3 lysine 9 dimethylation (H3K9me2) occupancy at the *Chat* promoter (2.3-fold, *t*[11] = 2.3, *p* = 0.044, Welch’s *t* test), *Chat* promoter CpG island (1.5-fold, *t*[11] = 2.2, *p* = 0.048, Welch’s *t* test), *Lhx8* promoter (2.1-fold, [10.6] = 2.2, *p* = 0.052, Welch’s *t* test), *Trka* promoter (1.8-fold, *t*[18] = 2.2, *p* = 0.020, Student’s *t* test), and *Trka* promoter CpG island (2.1-fold, *t*[18] = 3.0, *p* = 0.004, Student’s *t* test) relative to CONs (*n* = 10 wells/group). **B** ChIP assessment revealed that direct application of EtOH to FSC media significantly increased REST occupancy at the *Chat* promoter (2.7-fold, *t*[14.9] = 3.3, *p* = 0.005, Welch’s *t* test) and Lhx8 promoter (2.5-fold, *t*[11.9] = 2.2, *p* = 0.051, Welch’s *t* test) relative to CONs (*n* = 12 wells/group). Data are presented as mean ± SEM. ChIP analyses were run in duplicate. *p < 0.05, **p < 0.01.

We next investigated epigenetic gene repressive marker occupancy at cholinergic gene promoters and promoter CpG islands located near promoter transcription start sites in the FSC model to provide insight into EtOH-induced gene repression. We report EtOH increased H3K9me2 occupancy approximately 2.3-fold at the *Chat* promoter and approximately 1.5-fold at the *Chat* promoter CpG island. Similarly, EtOH increased H3K9me2 occupancy approximately 2.1-fold at the promoter region of the cholinergic lineage gene *Lhx8*. Finally, EtOH treatment increased H3K9me2 occupancy approximately 1.8-fold at the *Trka* promoter and approximately 2.1-fold at the *Trka* promoter CpG island (Fig. 4A). Importantly, H3K9me2 occupancy was unchanged at the *Parvb* promoter (Supplementary Fig. 3C). Thus, EtOH increases H3K9me2 occupancy at *Chat*, *Lhx8*, and *Trka* gene promoters but not *Parvb*, consistent with epigenetic repression of key enzymatic, lineage, and NGF trophic receptors of the BFCN phenotype.

The *Chat* and *Lhx8* gene promoters contain the consensus 21-base-pair DNA RE1 binding sequence that binds the transcriptional repressor REST, and REST is known to regulate expression of *Chat* and other cholinergic genes [54]. We report that EtOH treatment increased REST occupancy at the *Chat* promoter approximately 2.7-fold. Although *Trka* does not contain the RE1 binding site, we did observe an approximate 2.5-fold increase of REST occupancy at the cholinergic lineage gene *Lhx8* promoter (Fig. 4B). Thus, EtOH increases REST occupancy at *Chat* and *Lhx8* promoter regions, consistent with repression of BFCN phenotype genes.

**Fig. 5:**
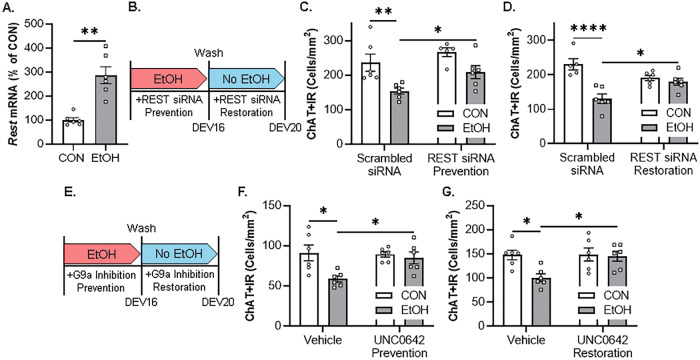
Ex vivo EtOH-induced increase of REST-G9a epigenetic signaling in the basal forebrain leads to reversible loss of ChAT+IR basal forebrain cholinergic neurons (BFCNs). **A** Real-time PCR (RTPCR) analysis revealed ex vivo EtOH application increased *Rest* mRNA approximately 2.9-fold in the FSC model relative to CONs (*t*[5.8] = 5.1, *p* = 0.003, Welch’s *t* test; *n* = 6 wells/group). **B** Schematic depicting experimental design for ex vivo EtOH REST siRNA prevention and restoration experiments. For the prevention experiment, FSCs were treated with EtOH (100 mM; 96 h) in combination with REST siRNA or vehicle until tissue collection. For the restoration experiment, FSCs were treated with EtOH (100 mM; 96 h), slices washed to remove EtOH on day ex vivo (DEV) 16, and then treated with REST siRNA or vehicle for 96 h until tissue collection on DEV20. **C** Modified unbiased stereological assessment revealed that direct application of EtOH to FSC media caused a 30% (±4%) reduction of ChAT+IR BFCNs relative to CONs (*t*[8] = 3.4, *p* = 0.009, Student’s *t* test), an effect that was prevented by treatment with REST siRNA (*t*[8] = 2.4, *p* = 0.043, Student’s *t* test; *n* = 5 wells/group). **D** Modified unbiased stereological assessment revealed that direct application of EtOH to FSC media caused a 43% (±6%) reduction of ChAT+IR neurons relative to vehicle-treated FSCs (*p* = 0.00001, Tukey’s HSD), an effect that was restored (*p* = 0.039, Tukey’s HSD; *n* = 5-6 wells/group) with the REST siRNA (interaction of 2 × 2 ANOVA: *F*[1,19] = 13.7, *p* = 0.0015). **E** Schematic depicting experimental design for ex vivo EtOH G9a inhibitor prevention and restoration experiments. For the prevention experiment, FSCs were treated with EtOH (100 mM; 96 h) in combination with the G9a inhibitor UNC0642 (1.0 μM; 96 h) or vehicle until tissue collection. For the restoration experiment, FSCs were treated with EtOH (100 mM; 96 h), slices washed to remove EtOH on DEV16, and then treated with the G9a inhibitor UNC0642 (1.0 μM; 96 h) or vehicle until tissue collection on DEV20. **F** Modified unbiased stereological assessment revealed that direct application of EtOH to FSC media caused a 36% (±4%) reduction of ChAT+IR BFCNs relative to CONs (*p* = 0.011, Tukey’s HSD), an effect that was prevented (*p* = 0.050, Tukey’s HSD; *n* = 6 wells/group) by treatment with UNC0642 (interaction of 2 × 2 ANOVA: *F*[1,20] = 4.5, *p* = 0.046). **G** Modified unbiased stereological assessment revealed that direct application of EtOH to FSC media caused a 32% (±5%) reduction of ChAT+IR neurons relative to vehicle-treated FSCs (*p* = 0.024, Tukey’s HSD), which was restored (*p* = 0.035, Tukey’s HSD; *n* = 6 wells/group) by treatment with the G9a inhibitor UNC0642 (interaction of 2 × 2 ANOVA: *F*[1,20] = 4.3, *p* = 0.052). RTPCR analyses were run in duplicate. Data are presented as mean ± SEM. *p < 0.05, **p < 0.01, ***p < 0.001, ****p < 0.0001.

Similar to the in vivo rat and post-mortem human experiments, application of EtOH in the FSC model caused an approximate 2.9-fold increase of *Rest* mRNA (Fig. 5A). We next determined if knockdown of REST in the FSC model using a REST siRNA cocktail that was previously reported to decrease *Rest* expression [13], prevented the EtOH-induced loss of ChAT+IR BFCNs (Fig. 5B). Although we did not observe a significant interaction, EtOH alone caused a 30% (±4%) reduction of ChAT+IR BFCNs, consistent with earlier experiments, an effect that was prevented by co-administration of the REST siRNA cocktail (Fig. 5C). As these data reveal that the EtOH-induced reduction of ChAT+IR BFCNs involves REST, we next assessed if this loss is reversible in the FSC model. FSCs were treated with EtOH for 96 h as before to allow for loss of ChAT+IR BFCNs; then, after EtOH treatment, REST siRNA was applied to culture media for 96 h following removal of EtOH (Fig. 5B). We found that EtOH caused a 43% (±6%) loss of ChAT+IR BFCNs that persisted after EtOH removal for 96 h, and REST siRNA restored the loss of ChAT+IR BFCNs (Fig. 5D). We next assessed whether blockade of the methyltransferase G9a prevents the EtOH-induced loss of ChAT+IR BFCNs (Fig. 5E). We report that EtOH caused a 36% (±4%) reduction of ChAT+IR BFCNs, an effect that was prevented by co-administration of the G9a inhibitor UNC0642 (1.0 μM; Fig. 5F). We next determined if post-EtOH application of UNC0642 would restore the loss of ChAT+IR BFCNs. We found that EtOH caused a 32% (±5%) reduction of ChAT+IR BFCN that persisted for 96 h following removal of EtOH, and blockade of G9a with UNC0642 restored the EtOH-induced loss of ChAT+IR BFCNs (Fig. 5G). Thus, these data support EtOH induction of REST and the methyltransferase G9a as contributing to reversible epigenetic repression of the BFCN phenotype. Taken together, these data support EtOH induction of REST and the methyltransferase G9a as contributing to increases in H3K9me2 and REST occupancy at cholinergic gene promoters that repress the cholinergic neuron phenotype.

## Discussion

**Fig. 6:**
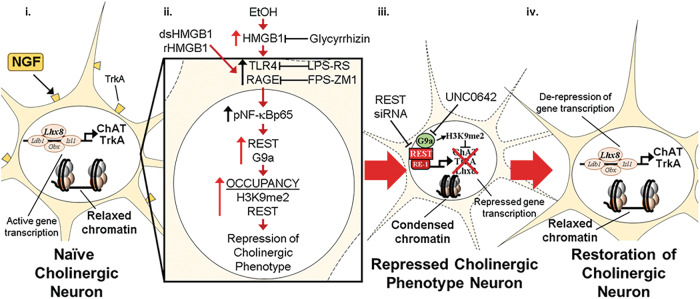
Schematic depicting the proposed neuroimmune-epigenetic mechanism underlying EtOH-induced epigenetic repression of the basal forebrain cholinergic neuron (BFCN) phenotype. **A** In the naïve basal forebrain, healthy BFCNs express the cholinergic linage gene LIM/homeobox protein 8 (Lhx8), the ACh-synthesizing enzyme ChAT, the high-affinity NGF receptor tropomyosin receptor kinase A (TrkA), and other cholinergic phenotype genes. Schematic depicting relaxed, open chromatin allowing active transcription of cholinergic genes and maintenance of the cholinergic phenotype. The *Chat* and *Lhx8* gene promoters contain the consensus 21-base-pair DNA binding sequence RE1 [54, 64]. Note that the transcription factor RE1-silencing transcription factor (REST) is not bound to the RE1 binding site allowing relaxed chromatin and active transcription of *Chat*, *Trka*, *Lhx8*, and other cholinergic genes in healthy BFCNs. **B** Schematic depicting EtOH induction and release of high-mobility group box 1 (HMGB1), which is an endogenous agonist at Toll-like receptor 4 (TLR4; disulfide HMGB1 [dsHMGB1]) and the receptor for advanced glycation end products (RAGE; fully reduced HMGB1 [rHMGB1]). Ethanol causes extracellular release of HMGB1, which binds to TLR4 and RAGE on glia and BFCNs [14], leading to nuclear translocation and activation of the transcription factor NF-κB p65 in BFCNs [13, 14]. The HMGB1 inhibitor glycyrrhizin and the RAGE antagonist FPS-ZM1 blocked whereas the TLR4 antagonist LPS-RS blunted the HMGB1-induced loss of ChAT+IR BFCNs. NF-κB transcriptional activation involves formation of the canonical p65/p50 heterodimer that not only activates proinflammatory gene transcription but also forms repressome complexes of HMGB1-G9a [72, 73]. Ethanol induces the transcriptional repressor REST and the histone 3 lysine 9 (H3K9) methyltransferase G9a that parallels increased occupancy of REST and the repressive marker H3K9 dimethylation (H3K9me2) on cholinergic phenotype genes culminating in condensed, closed chromatin and repression of the cholinergic phenotype. REST is known to regulate cholinergic gene expression during development [54], and REST binding to RE1 leads to recruitment of G9a, which represses gene transcription through dimethylation of H3K9 [52, 53]. Inhibition of G9a with UNC0642 and REST with REST siRNA prevent and restore the EtOH-induced loss of ChAT+IR BFCNs. Interestingly, HMGB1 interactions with NF-κB are linked to H3K9me2 through recruitment of the methyltransferase G9a to repress gene transcription [49–51]. **C** Schematic depicting restoration of condensed chromatin to a relaxed, open chromatin state allowing de-repression and active transcription of cholinergic genes and restoration of the FCN phenotype.

The current study extends prior in vivo studies linking proinflammatory neuroimmune and epigenetic repressive mechanisms to the persistent, but reversible, AIE-induced loss of ChAT+IR BFCNs [14–16]. We previously reported unique sensitivity of adolescent cholinergic neurons to binge drinking models as identical intermittent binge ethanol exposure in adults does not affect cholinergic neuron populations [8]. Indeed, it was reported 28 weeks, but not shorter intervals, of sole-source alcohol liquid diet in adulthood is required to reduce ChAT+ BFCNs [55, 56]. In the post-mortem human brain of individuals with AUD, expression of HMGB1, TLR4, and RAGE are increased whereas markers of cholinergic neurons are reduced [8, 10, 12, 57]. In the preclinical AIE model, exercise, indomethacin, and galantamine treatment post-AIE reverse proinflammatory neuroimmune induction and restore the loss of ChAT+IR BFCNs [14, 15, 58]. While these in vivo studies link proinflammatory HMGB1 neuroimmune signaling to the persistent, but reversible, AIE-induced loss of BFCNs, mechanistic studies are needed to directly link these changes. In the current study, our laboratory utilized an organotypic FSC model to investigate the mechanisms underlying EtOH-induced reversible epigenetic repression of the BFCN phenotype. We report that EtOH treatment of FSCs decreased populations of ChAT+IR BFCNs and caused somal shrinkage of the remaining ChAT+ neurons that was accompanied by reduced expression of multiple cholinergic phenotype genes, similar to the in vivo AIE model. Both models express the known cholinergic trophic factor receptors *Trka* and *Ngfr* [13] as well as key cholinergic phenotype genes (*Ache*, *Chat)* and the cholinergic lineage transcription factor *Lhx8*. Further, *Chrna7* mRNA is similarly reduced, whereas increases in *Hmgb1*, *Il1b* and *Ccl2* are observed across both models. These studies suggest that our FSC model mimics in vivo AIE, allowing direct studies on HMGB1 signaling. Previous studies in hippocampal slice culture reported active cellular release of HMGB1 stimulated by EtOH as well as glutamate, HDAC inhibitors, neuronal activation, and TLR4 agonizts [27, 59, 60]. We report here that EtOH treatment of FSCs induces *Hmgb1* mRNA that is accompanied by increased media levels of HMGB1. Release of HMGB1 involves acetylation of HMGB1 that modulates nuclear translocation and subsequent release of HMGB1 that does not influence its redox state [27, 47]. Although we did not determine the redox form of HMGB1 released by EtOH, direct treatment with dsHMGB1 (TLR4 agonist) and rHMGB1 (RAGE agonist) reduced ChAT+IR BFCNs, while antagonists to TLR4 (i.e., LPS-RS) and RAGE (i.e., FPS-ZM1) blunted and blocked, respectively, the HMGB1-induced reductions of ChAT+IR BFCNs, respectively. This suggests that HMGB1 signaling through multiple receptors can reduce ChAT+IR, consistent with EtOH release of HMGB1 that activates TLR4 and RAGE receptors, thereby leading to the loss of ChAT+ BFCNs. This hypothesis is further supported by our finding that glycyrrhizin, an HMGB1 antagonist, blocked the EtOH-induced reduction of ChAT+IR BFCNs. HMGB1-TLR4/RAGE signaling is known to activate the nuclear transcription factor NF-κB and we previously reported that AIE and direct TLR4 stimulation with LPS in the FSC model increases basal forebrain expression of HMGB1, RAGE, TLR4, and phosphorylated NF-κBp65 as well as colocalization of phosphorylated NF-κBp65 in ChAT+IR BFCNs [13, 14], consistent with HMGB1 signaling directly on ChAT+ neurons. Taken together, these findings suggest HMGB1-TLR4/RAGE signaling on ChAT+ BFCNs leads to reduced expression of cholinergic phenotype markers as well as neuronal somal shrinkage (Fig. 6).

HMGB1 and proinflammatory neuroimmune signaling are associated with neurodegeneration, and studies reporting loss of neuron-specific markers, such as ChAT, are interpreted as indicative of cell death. However, our in vivo studies in adults following AIE did not find evidence of neuronal death or loss of neuronal NeuN protein or mRNA expression [15, 61]. We find no evidence of cell death in the ex vivo FSC model as measured using the cell death marker PI or a loss of neuronal NeuN protein or mRNA expression. In contrast, we have reported cell death in the hippocampal neurogenic niche that may reflect the proliferative milieu of the subgranular zone of the hippocampal dentate gyrus wherein newborn neurons that are not integrated into the existing hippocampal neurocircuitry undergo cell death, an effect that is exacerbated by AIE treatment [36, 62]. Further, anti-inflammatory treatments post-AIE restore ChAT+IR BFCN loss that is accompanied by reversal of repressive H3K9me2 occupancy on cholinergic gene promoters [14, 15]. The effects of EtOH on BFCN phenotype appear to be mediated, in part, through proinflammatory signaling as treatment with the TLR4 agonist LPS increases REST and G9a expression as well as occupancy of epigenetic repressive markers H3K9me2 and REST at cholinergic gene promoters in the absence of cell death [13]. We replicated these in vivo findings in our ex vivo FSC model as EtOH treatment increases REST expression as well as occupancy of the repressive marker H3K9me2 at *Chat*, *Trka*, and *Lhx8* gene promoters and REST at the *Chat* and *Lhx8* gene promoters, consistent with our in vivo studies suggesting epigenetic silencing mechanisms. In the FSC model, REST siRNA during EtOH exposure blocks loss of ChAT+IR, whereas REST siRNA after EtOH exposure restores ChAT+ BFCNs. Similarly, we report here that the G9a inhibitor UNC0642 both prevents and reverses EtOH-induced reductions of ChAT+IR BFCNs. While it is possible that knockdown of REST and G9a may have off target effects, our findings of increased REST and H3K9me2 occupancy at cholinergic gene promoters, coupled with reversal of ChAT+ neuron loss following blockade of REST and G9a strongly supports our proposed mechanism. These findings strongly support reversible REST recruitment of G9a increasing H3K9me2 repression of the cholinergic transcriptome, perhaps to reduce excitability to maintain neuron survival by reducing terminal differentiation of the cholinergic transcriptome and reducing somal size. Multiple replicates of the in vivo AIE model find a ChAT+IR BFCN loss of ~30% and somal size reductions of 15–25%, suggesting limits to ChAT+ loss through unknown mechanisms. This is supported by our FSC dose responses to EtOH, dsHMGB1-TLR4 and rHMGB1-RAGE that reduce ChAT+ neurons to a maximum of approximately 30%, with the combination being only slightly greater than either alone. Lipopolysaccharide (LPS) is a TLR4 agonist known to increase brain proinflammatory signaling both in vivo and ex vivo that reduces ChAT+IR BFCNs and the cholinergic transcriptomes approximately 20–30%, comparable to the ChAT+ loss observed in the in vivo AIE model and ex vivo EtOH FSC model [13, 14, 16]. However, the proinflammatory response is far greater with LPS than EtOH, consistent with a protective limit to proinflammatory reductions of ChAT+IR BFCNs [13]. The surprising resistance to further loss of ChAT+IR BFCNs supports a protective mechanism consistent with REST repression being a protective response to EtOH-induced increases in HMGB1-TLR4/RAGE neuroimmune signaling.

REST is a well-known transcriptional repressor that binds to RE1, suppressing expression of neuronal genes and regulating neuronal differentiation, including later stages of differentiation of excitatory and inhibitor synapses that establish stable neurocircuitry [63]. Acetylcholine is excitatory, and we find reversible REST repression of the cholinergic phenotype. The *Chat* and *Lhx8* genes contain the consensus REST repressor 21-base-pair DNA binding sequence RE1 [54, 64]. Lhx8 regulates multiple cholinergic genes [65], including the high-affinity NGF receptor TrkA [66] that is known to promote cholinergic differentiation as well as the survival and maintenance of BFCNs [67, 68]. An Lhx8-TrkA-NGF positive feedback loop is hypothesized to maintain BFCNs in their highly differentiated state [66]. REST repression of Lhx8 is consistent with the AIE- and EtOH-induced suppression of the cholinergic transcriptome and reduced neuronal excitability and perhaps diminished cerebral excitability. Reduced nuclear REST has been reported in several dementia disorders (e.g., AD) and is consistent with mouse conditional knockout studies reporting that REST deletion leads to age-related neurodegeneration [69]. REST is hypothesized to reduce excitability, protecting neurons since high levels of REST expression are found in the brain of humans cognitively intact at 90–100 years of age [70, 71]. Our findings of neuroimmune signaling inducing REST and loss of ChAT+IR BFCN phenotype may represent a neuroprotective mechanism to avoid neuronal death.

In summary, EtOH treatment of FSC recapitulates the in vivo AIE-induced loss of ChAT+IR BFCNs, ChAT+ neuron somal shrinkage, and reduction of cholinergic transcriptome. Ex vivo inhibition of EtOH-induced HMGB1 as well as dsHMBG1-TLR4 and rHMGB1-RAGE signaling in the FSC model blocked the loss of ChAT+IR BFCNs. EtOH induced the transcriptional repressor REST and the H3K9 methyltransferase G9a, and increased repressive H3K9me2 and REST occupancy at *Chat*, *Trka*, and *Lhx8* promoter regions, consistent with epigenetic repression of the cholinergic phenotype. REST siRNA and the G9a inhibitor UNC0642 both prevented and restored the EtOH-induced loss of ChAT+IR BFCNs. Together, these data reveal that EtOH exposure induces a novel neuroplastic process involving neuroimmune signaling and transcriptional epigenetic gene repression, resulting in the reversible loss of the cholinergic neuron phenotype (Fig. 6).

### Supplementary information

Supplementary Figure 1

Supplementary Figure 2

Supplementary Figure 3

Supplementary Table 1
