# Supplementary material for: HMGB1 neuroimmune signaling and REST-G9a gene repression contribute to ethanol-induced reversible suppression of the cholinergic neuron phenotype
Source: Mol Psychiatry. Author manuscript; Available in PMC 2024 Apr 25. (PMC10764639; doi:10.1038/s41380-023-02160-6)
Supplement: Supplemental Figure 3 [file NIHMS1934470-supplement-Supplemental_Figure_3.docx]

**Supplementary Figure 3. *In vivo* adolescent intermittent ethanol (AIE) and *ex vivo* EtOH do not affect populations of parvalbumin+IR GABAergic neurons in the basal forebrain.** (A) Modified unbiased stereological assessment revealed that AIE treatment did not change parvalbumin+IR GABAergic neuron populations in the adult (P80) basal forebrain relative to CONs (*t*(18)=1.12, *p*=0.276) n = 8/group. (B) Modified unbiased stereological assessment revealed that direct application of EtOH (100 mM; 96 h) to FSC media did not affect parvalbumin+IR GABAergic neuron populations relative to CONs (*t*(9)=0.904, *p*=0.389). (C) Chromatin immunoprecipitation revealed that direct application of EtOH (100 mM; 96 h) to FSC media did not affect H3K9me2 occupancy at the *Parvb* promoter relative to CONs (*t*(4)=0.205, *p*=0.847). Data are presented as mean ±SEM. ChIP analyses were run in duplicate. n = 3-6 wells/group.

B.

A.

C.
