# Supplementary material for: HMGB1 neuroimmune signaling and REST-G9a gene repression contribute to ethanol-induced reversible suppression of the cholinergic neuron phenotype
Source: Mol Psychiatry. Author manuscript; Available in PMC 2024 Apr 25. (PMC10764639; doi:10.1038/s41380-023-02160-6)
Supplement: Supplemental Table 1 [file NIHMS1934470-supplement-Supplemental_Table_1.docx]

| **Supplementary Table 1.** Summary of 2 × 2 ANOVA Results. | | | | | |
| --- | --- | --- | --- | --- | --- |
| Experiment | Figure | Comparison | df | *F*-value | *p*-value |
| EtOH +/- Glycyrrhizin | 2D | EtOH | 1,20 | 5.03 | 0.037 |
|  |  | Glycyrrhizin |  | 3.40 | 0.080 |
|  |  | EtOH × Glycyrrhizin |  | 20.94 | <0.001 |
| dsHMGB1 +/- LPS-RS | 2F | dsHMGB1 | 1,20 | 7.87 | 0.011 |
|  |  | LPS-RS |  | 3.48 | 0.077 |
|  |  | dsHMGB1 × LPS-RS |  | 0.81 | 0.377 |
| EtOH +/- REST siRNA Prevention | 5C | EtOH | 1,16 | 14.47 | 0.002 |
|  |  | REST siRNA |  | 11.55 | 0.004 |
|  |  | EtOH × REST siRNA |  | 0.06 | 0.811 |
| EtOH +/- REST siRNA Restoration | 5D | EtOH | 1,19 | 21.07 | <0.001 |
|  |  | REST siRNA |  | 0.12 | 0.731 |
|  |  | EtOH × REST siRNA |  | 21.07 | 0.002 |
| EtOH +/- UNC0642 Prevention | 5F | EtOH | 1,18 | 10.34 | 0.005 |
|  |  | UNC0642 |  | 2.26 | 0.149 |
|  |  | EtOH × UNC0642 |  | 2.77 | 0.113 |
| EtOH +/- UNC0642 Restoration | 5G | EtOH | 1,20 | 5.76 | 0.026 |
|  |  | UNC0642 |  | 4.60 | 0.045 |
|  |  | EtOH × UNC0642 |  | 4.26 | 0.052 |
