# Supplementary material for: HMGB1 neuroimmune signaling and REST-G9a gene repression contribute to ethanol-induced reversible suppression of the cholinergic neuron phenotype
Source: Mol Psychiatry. Author manuscript; Available in PMC 2024 Apr 25. (PMC10764639; doi:10.1038/s41380-023-02160-6)
Supplement: Supplemental Figure 2 [file NIHMS1934470-supplement-Supplemental_Figure_2.docx]

**Supplementary Figure 2. *Ex vivo* EtOH dose response and time course ChAT+IR basal forebrain cholinergic neuron (BFCN) loss in the rat basal forebrain slice culture model.** (A) Application of EtOH at a concentration of 50 mM (*p*=0.001, Dunnett’s test) and 100 mM (*p*=0.004, Dunnett’s test), but not 10 mM (*p*=0.060, Dunnett’s test) or 30 mM (*p*=0.592, Dunnett’s test), significantly decreased ChAT+IR BFCNs relative to CONs (*F*[4,24]=6.1, *p*=0.002, one-way ANOVA). (B) Time course analysis revealed that EtOH (100 mM) significantly decreased ChAT+IR BFCNs at 24 h (*p*=0.002, Dunnett’s test), 48 h (*p*=0.004, Dunnett’s test), and 96 h (*p*=0.0002, Dunnett’s test) relative to CONs assessed at 96 h (*F*[3,20]=9.7, *p*=0.0004, one-way ANOVA). Data are presented as mean ±SEM. ** *p*<0.01, *** *p*<0.001, relative to Veh conditions. n = 5-6/group.

A.

B.
