# Supplementary material for: HMGB1 neuroimmune signaling and REST-G9a gene repression contribute to ethanol-induced reversible suppression of the cholinergic neuron phenotype
Source: Mol Psychiatry. Author manuscript; Available in PMC 2024 Apr 25. (PMC10764639; doi:10.1038/s41380-023-02160-6)
Supplement: Supplemental Figure 1 [file NIHMS1934470-supplement-Supplemental_Figure_1.docx]

**Supplementary Figure 1. *Ex vivo* EtOH dose response for neuronal NeuN+IR and time course analysis of the cell death marker propidium iodide (PI) in the rat basal forebrain slice culture model.** (A) Application of EtOH at a concentration of 50 mM and 100 mM did not affect expression of the neuronal marker NeuN relative to CONs (*F*[2,15]=0.36, *p*=0.703, one-way ANOVA). (B) Time course analysis revealed that EtOH (100 mM) did not increase cell death as assessed using PI at 6h, 24h, or 96h of EtOH treatment relative to time-matched CONs (*F*[3,20]=0.56, *p*=0.647, one-way ANOVA). N = 6/group. Data are presented as mean ±SEM.

B.

A.
